# Factors Affecting Engagement in Screening Clinics; Exploring the Experiences of Patients with Rare Endocrine Gene Disorders

**DOI:** 10.1177/23743735251316120

**Published:** 2025-03-16

**Authors:** Samia Elyoussfi, Priscilla Harries, Meriel Norris, Lorraine DeSouza, William Drake

**Affiliations:** 1 Department of Health Sciences, Brunel University alumni, London, UK; 2 Research, Business and Innovation, Kingston University London, Head of Graduate Research School and Researcher Development, London, UK; 3 3890 Brunel University London; 4 Department of Endocrinology, St Bartholomew’s Hospital

**Keywords:** Rare endocrine syndromes, patient experience, patient engagement, screening clinics

## Abstract

The aim was to explore the patient experience of those attending screening appointments for rare endocrine syndromes. Obtaining insights into the factors that potentially enhanced or detracted from attendance and engagement with the clinics could assist in developing strategies to promote patient engagement. A qualitative approach using semi-structured interviews was employed to understand individuals’ perceptions and experience of the screening clinics. Twelve interviews were conducted with patients (age 10-66 years, purposive sampling). Four main themes were identified: (1) Perception at a distance, (2) Seeing my future self (3) The body and person in clinic and (4) The patient or doctor, who knows best? These highlighted several areas which could be used to inform approaches to promote enhanced patient engagement: the importance of careful management of projections of self, balancing information overload and honesty, interpersonal relationships and humanisation of care and assisting with the early navigation for the non-expert individual.

## Introduction

Rare endocrine syndromes such as von-Hippel Lindau Syndrome (VHL), multiple endocrine neoplasia (MEN) and mutations in the succinate dehydrogenase complex (SDHx) can lead to the development of tumours^1^ that can impact on patients’ quality of life and morbidity. Such rare endocrine syndromes require extensive screening and surveillance; the manifestations of the syndromes are multifaceted^2^ and may or may not be symptomatic. Screening is important considering that with the combination of advanced surgical and radiation oncology techniques, the morbidity and mortality of patients with rare endocrine syndromes like VHL can be significantly reduced.^3^ Despite the evidence-based guidance on screening, little is known about how individuals engage with and experience the process of screening and surveillance. This is important to understand, as generally, adherence to screening is reported as low.^4^

The patient experience is described as consolidating the patients’ perspective regarding the health care they receive^5,6^; understanding the patient perspective of their experience may aid in the understanding of the patients’ experience.^7^ Patient engagement is suggested as the ability to decide to partake in care in a way that is appropriate to the individual in collaboration with a healthcare provider.^8^ Some experiences may be specific for the rare disease patient. For instance, patients affected by various rare diseases such as Marfan syndrome described feeling not adequately informed about their diagnosis.^9^ Moreover, patients with diverse rare diseases conveyed high levels of anxiety and depression symptoms.^10^

The role of the rare endocrine screening clinics is to provide regular surveillance for mutations and treatment options such as surgery and radiation oncology for when such tumours form. These rare syndromes are generally overseen by specialist national referral centres. Patients generally have a review appointment at least once a year. As missed appointments reduce the potential to deliver the optimal provision of genetic health services^11^ it was necessary to understand how best to support patient engagement in the screening and surveillance clinics.

The aim of this study was two-fold: (1) to explore patients’ experiences and perceptions of attending the rare endocrine syndromes screening clinic and (2) to understand their views of the factors affecting their engagement.

## Method

### Study Design

The objectives focused on capturing the meaning patients ascribe to their experience of the screening clinics, thus making a qualitative design suitable to answer questions pertaining to the viewpoint of the participant.^12^ Semi-structured interviews were used as they support the development and examination of individuals’ experiences.^13^

The Consolidated Criteria for Reporting Qualitative Research (COREQ) guidelines were used^14^ to guide the development and reporting of this study.

### Sampling and Recruitment

This study used a purposive sampling strategy due to the information participants needed to provide about their screening clinic experience.^15^ Patients were recruited from the rare endocrine screening clinics. Inclusion criteria were that the patients were registered at, and attending, the endocrine screening clinic, diagnosed with a rare endocrine syndrome (SDH/MEN/VHL) and had the capacity to provide informed consent or assent for children, as determined by use of English language. Exclusion criteria included issues of risk identified by the clinical team, such as upcoming surgical operation. The recruitment period was between the 26^th^ June 2018 and 6^th^ September 2018. Adults and children who expressed an interest in participation provided their own or their guardian's phone number and were given the appropriate patient information sheets (PIS). Following receipt of the PIS an in-person interview was arranged. Informed written consent was taken from adult participants before the interview commenced. For children, each assented and their parent/guardian then provided written informed consent.

### Interviews

Interviews were carried out between 4^th^ July 2018 and 8^th^ September 2018 and were conducted at each patient's chosen place. A topic guide was designed jointly by the authors (Supplemental material 1). Clinical preliminary observations and the study aims were used to provide broad areas for the interview topic guide. Literature and preliminary clinical observations informed the development of the topic guide. The final topic guide was piloted; due to the challenge of accessing rare syndromes participants, this was done within the authors and role play. It included: the patient's experience of living with their condition; their experiences of using the screening clinic, what they understood to be the offered provision, factors that influenced engagement and how these impacted on how they used the services, and how the services could be developed to best support them. Each interview was digitally recorded and later transcribed verbatim.

### Data Analysis

The interview transcripts were analysed through an inductive approach using thematic analysis as documented by Braun and Clarke.^16^ Thematic data analysis develops themes across the whole data corpus^17^ and can be used when making sense of people's lived experiences.^18^

After transcription analysis was initiated with further data familiarisation^19^ through re-reading the transcripts and extracts of important text were highlighted and coded within NVivo version 12. Codes were then collated into categories, sub-themes and finally into candidate themes.^16^ The lead researcher carried out the data analysis while three additional authors confirmed the categorisation, to verify trustworthiness.^20^ A strategy that was used to address reflexivity was the use of a reflexive diary throughout the course of the participant interviews.

## Results

Twelve patients volunteered and completed interviews. Table 1 details the demographic profile of the patients; pseudonyms are used in reporting to protect confidentiality. Four main themes (with subthemes) were developed from the data: (1) perception at a distance, (2) seeing my future self (3) the body and person in clinic and (4) the patient or doctor, who knows best? Table 2 shows the main themes and the explanation of the theme. Table 3 provides quotes, with age, gender and syndrome in italics; ellipses are used to reflect unrelated text.

**Table 1.** Interview Patients’ Demographic Profiles.

Patient pseudonym	Age decade	Gender	Diagnosis	Length of time using the service
Emma	60s	Female	SDH	15 years
Olivia	40s	Female	SDH	16 years
Abigail	60s	Female	SDH	2 years
Elsa	50s	Female	MEN	43 years
Emily	10s	Female	MEN	8 years
Grace	10s	Female	MEN	10 years
Andrew	30s	Male	MEN	30 years
David	50s	Male	VHL	26 years
Jacob	50s	Male	VHL	29 years
Connor	10s	Male	VHL	2 months
Nolan	60s	Male	MEN	36 years
Miles	50s	Male	VHL	20 years

Key: MEN- Multiple endocrine neoplasia, VHL- von Hippel-Lindau, SDH- mutations in the succinate dehydrogenase gene complex.

**Table 2.** Main Themes and Explanation.

Main themes	Explanation of themes
Theme (1) Perception at a distance	This theme encapsulated how the patients regarded the screening service when they are out of the clinic.
Theme (2) Seeing my future self	This theme explores what influences the level of engagement with the screening clinics.
Theme (3) The body and person in clinic	This theme focuses on the patients’ experiences in clinic and how these may enhance or deter clinic attendance*.*
Theme (4) The patient or doctor, who knows best?	This theme focuses on the development and expectation of patient expertise.

**Table 3.** Main Themes and Subthemes with Quotations.

Theme (1) Perception at a distance
**Subtheme 1. Out of sight, out of mind** “It's like throughout the year, I won't think about (clinic) or the MEN (her diagnosis) won't get thought about or it will be a glancing, fleeting thought…so out of sight, out of mind.” (Elsa, 50s, F, MEN). “Well I’m not the worrier. You’ve just missed him (her husband), the worrier…Yes, you see I don't worry…until they (the doctors) tell me what the bottom-line of something is…” (Emma, 60s, F, SDH). **Subtheme 2. Anticipating the hospital visit** “When the letter comes through because obviously it's stamped on the envelope [details omitted for double-anonymized peer review] ….I start thinking…it's with the anxiety I feel that I don't want to be shut in or because obviously going into the tunnel now it's a bit like horrible”(Olivia, 40s, F,SDH). “So another problem is the fact that I’ve got to take a day off work to go all the way up to [details omitted for double-anonymized peer review]…So it's two days’ annual leave that I have to use up…(Elsa, 50s, F, MEN). “We’ve analysed this, and I said, it's two days out of 365 and it costs me £20 to go up there, get the results…You can ask any questions you want to ask..”(Emma, 60s, F, SDH).
**Theme (2) Seeing my future self**
**Subtheme 1. Snapshot of the future** “When I went there (support group)…There were people couldn't work…And I thought, bloody hell…It made me sort of think, oh God, I’m, you know. But some of the people there were quite relieved talking to me because they felt as though, oh, it's not that desperate a situation. But after that I didn't go actually, no.”(Nolan, 60s, M, MEN) “Touch wood, the way things are, medical science has improved so rapidly within this research – I know I’ve got two cousins who have survived, so I’m fully aware that it is not the death sentence that it used to be” Elsa, 50s, F, MEN). **Subtheme 2. Family matters** “I think it's a positive ultimately because having the family there… any issues that come up…we’re all aware of…my mum's had a few issues.. which if you were to sit there saying oh yes, you’ve got this, you’ve got this, you’ve got this.. it could worry the girls at their age, whereas the older they get the more they realise that yes okay, that condition could occur but because they’re monitoring it it's not a bad thing” (Andrew, 30s, M, MEN). “…We are going in now as all three of us went in together. I sometimes say is that a good idea? What if the children want to ask something that they don't want me to know or vice versa…but seeing that we’re a close family anyway…I’ll tell them when I came home anyway” (Emma, 60s, F, SDH). “Well, he (son) does understand it because his aunt died of it. But he just seems to think he's just one of them people who's invincible. He's young and he thinks, yes, I’m all right, nothing wrong with me…” (Miles, 50s, M, VHL)
**Theme (3) The body and person in clinic**
**Subtheme 1. Disembodiment** “..We are used to it (attending screening appointments), but I think it's a good idea. It's like getting a bit of MOT.” (Emma, 60s, F, SDH). “…It's a good way of spreading people out if they all arrive at once and dealing with it like a restaurant I suppose. Make them wait over there, then you move them there, then you move them in.” (Nolan, 60s, M, MEN) “…(during the MRI scan)Yes, so I’m just picturing I’m on the beach somewhere in Barbados…so they make it quite fun and relaxing for me because as soon as I go in they see I suffer from anxiety” (Olivia, 40s, F, SDH). **Subtheme 2. The balance between trust and scepticism** “Yes, I trust them, they know us, we know them really. No, I feel quite happy …” (Abigail, 60s, F, SDH). “We’ve got to know them. It's just like going to see your mate, because you’ve seen them so many times over the years. They’re not strangers, you can tell them anything you want” (Miles, 50s, M,VHL). “The people who use the toys to distract you (when dealing with needles)” (Emily, 10s, F, MEN). “…The guy, the radiographer said we only got a minute to finish (during the MRI scan). I said no I can't take any more because my back is hurting. I told them, the first place they lied to me I'm only going to be in for 15, 25 min, I've been in the machine for two hours…” (Jacob, 50s, M, VHL). “Years ago when I was first diagnosed I did used to try and look things…I ended up just frightening myself to death. And then ended up sometimes getting an earlier appointment and thinking, hang on, they’ve been telling me a load of lies here…And then I’d go up and see the Professor and he’d put my mind at rest… After that I’d given up looking things up. If there's something I really want to know I’ll ask the doctors.” (Nolan, 60s, M, MEN).
**Theme (4) The patient or doctor, who knows best?**
**Subtheme 1. Weight of expertise** “…I thought no, the doctor knows best, he's said it's probably fatty tissue and that's probably all it is, and sure enough that's what it was. So by having the knowledge that they know what they’re doing and know what they’re on about…” (Andrew, 30s, M, MEN). “Knowing that the doctors know what they’re on about, I guess” (Grace, 10s, F, MEN). “…They couldn't find anything. It's more than one year they try to find,.. Still I'm using the blood tablets now and I'm feeling tired, and I know haemoglobin is down…I'm feeling down, I'm like a doctor now. I have too much experience of my life” (David, 50s, M, VHL). **Subtheme 2. Assumed expertise** “It's just expecting me to ask something when I’m not provided with enough information to ask things. It's a bit like going into an exam without any revision, you don't know what you’re being asked” (Connor, 10s, M, VHL). “Well, I haven't really been provided with any information. All the information about the condition I’ve done myself by research from the internet” (Connor, 10s, M, VHL). “Information, definitely. If I know more about something that I have, then I can deal with it better” (Connor, 10s, M, VHL).

### Main Themes and Subthemes

#### Theme (1) Perception at a Distance

In the subtheme *Out of sight, out of mind* patients commented on how generally they do not think of their diagnosis or their appointment throughout the year; Elsa would at most briefly think of her diagnosis. Emma is similarly unconcerned and explains that her husband instead is the worrier, however, this was a suspended concern, which had the potential to emerge dependent on what the doctors tell her.

Others recounted in the subtheme *Anticipating the hospital visit* that closer to the time of their screening clinic appointment, an apparent shift is created, which brings the reality of their diagnosis to mind. Olivia conveyed that on receiving the appointment letter feelings of anxiety were brought to the forefront, prompted by concerns with symptoms and clinical tests. In addition to the emotional response, patients conveyed practicalities that needed to be managed in response to a clinic appointment. Elsa communicated the challenges of arranging annual leave, thus bringing the screening into a very practical reality. Conversely, there were some patients who recognised the worthwhile aspects of paying the travel costs and having a full day at the clinic; Emma explained how she had the chance to ask the doctors about anything.

#### Theme (2) Seeing my Future Self

In the subtheme *Snapshot of the future*, patients reiterated having glimpses of their future self with respect to their diagnosis prompted by attendance where they engage with others with similar conditions. Nolan illustrated how, during the first- and only-time attending support groups, he began to compare his state of diagnosis to others in the group and vice versa. Conversely, Elsa explained that observing how medical research has evolved over time and witnessing the survival of her cousins has given her a positive outlook.

Patients outlined in the subtheme *Family matters* how multigenerational family clinics and intergenerational knowledge are managed between family members. Andrew illustrated how this allowed for full disclosure of information and provided support, but also benefited the children by providing comfort to their potential worry. In contrast, Emma questioned the idea of family clinics, as she expressed hesitation to others being present, but at the same time continued to attend as a family. Examples were given where family members chose not to attend; Miles discussed how his son does not attend the clinics, despite experiencing the death of his aunt from the same diagnosis he has.

#### Theme (3) The Body and Person in Clinic

In the subtheme *Disembodiment* patients illustrated examples of disembodying practices which they saw as positive or appropriate coping methods. Emma used the metaphor of undergoing a MOT (Ministry of Transport test) positively to refer to the screening process. Similarly, Nolan recognised the benefits of undergoing such procedures in one day; however, his response displayed a sense of being a passive recipient with not much control over what happens in the clinic, where he is the object being moved from A to B etc The sense of splitting your physical form, from your embodied self was also echoed; Olivia described how her imagining she is on a beach can remove her from anxiety created by the discomfort she experienced in the MRI (magnetic resonance imaging).

The dynamic interrelationships of trust and mistrust between the clinic's staff and patients are portrayed in the subtheme *The balance between trust and scepticism.* Patients such as Abigail discussed the importance of interpersonal interactions which were fundamental to a sense of trust. This was supported by the familiarity created, as described by Miles, by virtue of the on-going history of the syndrome and visits. Whilst a younger patient, Emily, confirmed that the staff try to make the children more comfortable. Contrary examples illustrated how that trust could be disrupted, for instance Jacob by his previously perceived negative MRI scan experience, or increased suspicion expressed by Nolan regarding not receiving full information from the staff prompted by self-education.

#### Theme (4) The Patient or Doctor, Who Knows Best?

The subtheme *Weight of expertise* illustrates the responsibility towards the management of diagnosis. Some patients have complete reliance on the opinion of the medical staff; Andrew conveyed a sense of trust in the doctor's expertise. The portrayed expertise of the doctors at the clinic was also appreciated by younger patients; this seemed to be an important aspect to Grace when attending the clinic. In contrast, David discussed self-responsibility and due to the on-going history of the diagnosis he feels like a medical professional.

The subtheme *Assumed expertise* conveys the automatic expectation placed on some patients as expert patients. Due to the rare status label of the diagnosis, some recently diagnosed patients are unaware of the syndrome specifics; Connor illustrated this feeling in terms of being unprepared for an exam. Connor further describes how this sense of incomplete information led him to research the topic himself online. Moreover, he explained that he would be appreciative of such information as he felt the more informed regarding his diagnosis, the more he could manage the issues resultant from it.

## Discussion

This study provided a unique in-depth exploration of the patients’ experiences and perceptions of the rare endocrine syndromes screening clinics, which identified factors that potentially enhance or detract from attendance and engagement with the clinics.

### Factors Which Potentially Detract from Attendance and Engagement

Patients’ accounts included the avoidance of thoughts regarding their diagnosis and clinics; literature indicates that avoidance is a known strategy for people with chronic or incurable diagnoses.^21^ While patients reported not to worry on an everyday basis, significant others, in Emma's case her husband, did; this is an example of a ‘spillover’ effect of the diagnosis which can affect aspects of family members’ lives, from emotional health to quality of life.^22^

It has been recommended that appointment reminders to individuals can facilitate attendance.^23^ However, in this study, reminders triggered difficult memories; for example, Olivia's anxiety due to the letter could deter her attendance. Patients with rare diseases expressing anxiety symptoms are indicated in the literature.^10^ Previous studies reported that patients who are socially supported appeared to cope better with their diagnosis.^24^ However, Nolan's experience with the support group was not positive; this resonates with the social comparison theory; social comparison occurs between people with similar issues.^25^ Nolan's portrayal of possible anxiety while attending the patient support groups, is analogous with the findings of Palant and Himmel,^26^ who reported feelings of anxiety of patients when listening to others. Moreover, the feeling of anxiety with social groups may be a factor in the lack of clinic engagement, as patients are confronted with those diagnosed with same chronic illness. This issue was echoed by younger patients who chose not to attend with other family members if at all; young people miss more scheduled medical appointments than other age groups.^27^

Patients illustrated how they feel while attending the screening clinic, an interesting aspect is made by Emma, who used the analogy of undergoing a MOT (Ministry of Transport test), an annual test of vehicle safety in the United Kingdom. This analogy imparts a sense of the screening being a positive aspect, however, during the process they feel detached from their physical body through the description of their body as being mechanical and finely tuned, to some extent a mechanical person. Moreover, Nolan compared the clinic experience of undergoing several procedures in one day as being part of revolving seats in a restaurant. Therefore, he may consider himself to be part of a conveyer belt of patients just passing through the clinic, thus creating an impression of the patient being detached and not engaged in the screening clinics environment.

Patients with or at risk of rare endocrine syndromes expressed a lack of information when attending the screening clinic, this was also noted in a literature,^9^ where patients with rare diseases described having limited knowledge about their diagnosis. Individuals with rare diseases may face challenges that are different from those experienced in more common medical diagnoses^28^; Connor's explanation of the how information provides him a sense of empowerment as he ‘*can deal with it better’*. Therefore, reliable and up-to-date information is crucial for patients to be able to make informed choices about their diagnosis.^29^ Moreover, Muir^29^ discussed that nearly 70% of his study respondents with a rare syndrome did not feel that they were provided with sufficient information on their syndrome following diagnosis.

However, it was evident that for some too much information could result in unnecessary fear; Nolan describes how he researched some information regarding his diagnosis, however he *‘ended up just frightening’* himself and rescheduling his appointment to an earlier slot. Although obtaining information about his diagnosis increased Nolan's engagement, this was under the premise of anxiety that he was not given the correct information. This finding supports previous research where despite the probable benefits of accessible online health resources, some concerns have been expressed regarding possible anxiety-provoking effects of online medical information.^30^

### Factors Which Potentially Enhance Attendance and Engagement

Family networks were a positive factor in the level of engagement in the clinics; Andrew's anecdote of the importance of attending the clinics as a family is supported in the literature where immediate networks such as family and close friends were discussed to be of essential importance regarding mammography screening behaviour.^31^ Fear acted as a motivator but can be a barrier as known in mammography screening.^31^ Thereby having family members supporting each other in consultation, as illustrated by Andrew, may be a factor in the continuous engagement of some patients in the screening clinic. Patient and family-centred care is considered a key factor of high-quality care and has been increasingly linked to better health outcomes.^32^

In the literature the patient-provider relationship was rated second only to family relationships in level of importance to patients^33^; this is embodied by Miles who in this study refers to the experience of going to the clinic, as going to see his ‘*mate’,* thus conveying the ease and informality of interaction. Information exchange and fostering trusting relationships have been described as significant aspects of successful interpersonal communication between patients and care providers.^34^ High quality patient-provider communication has been demonstrated to correlate with longitudinal continuity of care,^35^ thus likely to influences patients’ interpersonal relationships with their healthcare providers and consequent screening clinic attendance.

An example of being detached in the screening clinic environment, is described by Olivia by illustrating that she envisions herself on a beach setting while undergoing her MRI scan. Such detachment is a possible coping strategy. Disassociating, as a form of coping with trauma, is echoed in the literature by Kennerly^36^ and Mollon.^37^ The processes depicted by patients such as daydreaming of a beach, is described as an out of body experience.^36^ Olivia notes that the team does make it relaxing; suggesting that creating an environment in which these personal strategies are supported is good practice.

In relation to behavioural maintenance factors, there is evidence that health anxious individuals will seek reassurance from sources such as the doctor,^38^ exemplified by Nolan, thereby increasing engagement. Those with health anxiety may be more inclined towards experiencing doctor disadvantages, thus could result in the use of alternative sources such as the internet,^39^ as illustrated by Connor. Consistent with this, interviews carried out by Singh et al^40^ demonstrated that health anxious students used the internet to filter problems before consulting doctors, therefore becoming ‘expert patients’.

The aim of this study was achieved in part, in that the interviews highlighted new insights into the patient experience, such as provision of patient information, which may assist in developing strategies to promote patient engagement. However, this study also conveyed areas that could be further explored such as the experience of younger patients and the patients who don't regularly attend appointments.

## Limitations and Strengths

Regarding limitations, this study included two child interviews which were short in length. Child interviews possess limitations as they can be a complex and challenging task.^41^ Future research could use other forms of interview methods, which include using pictures^42^; in addition to the use of specialised interview topic guides for children and young people.^43^ Additional limitations were in relation to the length of the adult interviews, which were short, however with the adult patients the topics were found to be covered effectively. The wide range of age: 10–66, is a limitation in terms of the difficulty in comparing children to adults; research with children is likely to differ from research with adults possibly due to the inherent difference between children, the perceptions of adults of children and the marginalised position that children hold in society.^44^ Further, the findings were not returned to the patients for comment; this could have been used to validate or assess the trustworthiness of the qualitative results.^45^

Strengths included recruitment of patients across all three screening clinics, which also included some children. Cooperation of the patient's willingness to convey individual experiences of the screening clinic was highly valued.

The study patients were recruited at a single tertiary referral centre, which the three different screening clinics are a part of; therefore, the patient views were from one geographical location. Moreover, this is a national referral centre for these rare syndromes, thus a suitable centre to develop models of best practice.

## Clinical Implications and Recommendations for Future Research

Potential implications include addressing issues of possible mental health of the patient with a diagnosis of a rare endocrine syndrome who are required to attend lifelong regular screening appointments. This was portrayed through the narration of instances of anxiety at the clinic and when outside of the clinical environment. The patient–service provider interpersonal relationship has been demonstrated to be significant in that it can affect whether the patient perception of the clinic is positive or negative. Therefore, recognition of the importance of the patient–service provider communication by the screening services is imperative; thus, appropriate communication skills training is necessary.

## Conclusion

This study identified themes based on patients’ experiences, which could be used to develop and promote regular patient engagement. The importance of careful management of projections of self, balancing information overload and honesty, interpersonal relationships and humanisation of care, and assisting with the early navigation for the non-expert individual were all highlighted.

## Supplemental Material

sj-docx-1-jpx-10.1177_23743735251316120 - Supplemental material for Factors Affecting Engagement in Screening Clinics; Exploring the Experiences of Patients with Rare Endocrine Gene Disorders Supplemental material, sj-docx-1-jpx-10.1177_23743735251316120 for Factors Affecting Engagement in Screening Clinics; Exploring the Experiences of Patients with Rare Endocrine Gene Disorders by Samia Elyoussfi, Priscilla Harries, Meriel Norris, Lorraine DeSouza and William Drake in Journal of Patient Experience

sj-docx-2-jpx-10.1177_23743735251316120 - Supplemental material for Factors Affecting Engagement in Screening Clinics; Exploring the Experiences of Patients with Rare Endocrine Gene Disorders Supplemental material, sj-docx-2-jpx-10.1177_23743735251316120 for Factors Affecting Engagement in Screening Clinics; Exploring the Experiences of Patients with Rare Endocrine Gene Disorders by Samia Elyoussfi, Priscilla Harries, Meriel Norris, Lorraine DeSouza and William Drake in Journal of Patient Experience
