# Supplementary material for: Factors Affecting Engagement in Screening Clinics; Exploring the Experiences of Patients with Rare Endocrine Gene Disorders
Source: J Patient Exp. 2025 Mar 16;12:23743735251316120. doi: 10.1177/23743735251316120 (PMC11912168; doi:10.1177/23743735251316120)
Supplement: sj-docx-1-jpx-10.1177_23743735251316120 - Supplemental material for Factors Affecting Engagement in Screening Clinics; Exploring the Experiences of Patients with Rare Endocrine Gene Disorders [file sj-docx-1-jpx-10.1177_23743735251316120.docx]

**Supplementary material 1**

**Patient interviews topic guide**

*Note on use of this topic guide:*

- We wish to encourage patients to discuss their experiences and views in an open way without excluding issues that may be of importance to individual participants and the study as a whole. Therefore, the questioning will be semi-structured and will be responsive to the respondents’ own circumstances, attitudes and experiences.

- The following guide does not contain pre-set questions but rather lists the key themes and sub-themes to be explored with each participant. This allows the interviewer to develop questions which are responsive to each individual participant. The topic guide does not include follow-up questions like ‘why’, ‘how’, 'when' etc. as it is assumed that participants’ contributions will be sufficiently explored throughout in order to understand why and how views, experiences and behaviours.

- Even though all topics will be explored with each participant, the order in which issues are addressed, as well as the amount of time spent on different themes will differ between participants.

**Introduction**

- Introduce self and thank the individual for agreeing to participate.

- Introduce research (funding, research design, outputs).

- Explain: confidentiality, tape recording, length of interview, nature of discussion (specific topics will addressed, but conversational in style, in your own words, no right or wrong answers), reporting and data storage/archiving. If you don’t want to discuss something I mention then just say so and we won’t talk about that. If you want to stop at any time just let me know and we will stop.

- Any questions.

- Obtain consent (written).

**1) Background-**

***Aim: to gather background contextual information which may have a bearing on experiences and can be followed up and explored during interview.***

- Personal circumstances (what and how long been diagnosed with, any additional family members with same diagnosis).

- How long been involved with the screening programme and service providers.

**2) Recent experience as a patient at the Barts Endocrine Screening programme-**

***Aim: to capture spontaneous reflections on the most recent experience and which aspects were important to participant.***

- Overall impression of the clinic and hospital.

- Experience (positive and negative aspects of experience).

- Satisfaction (how satisfied overall with care and treatment received).

**3) Awareness of clinic services-**

***Aim: to discover how patients understand the screening service.***

- What do they think is available.

- When/or at what stage have they used the service.

**4) Engaging with the screening programme-**

***Aim: in what manner do patients make decisions about using the service.***

- Decision making processes (any prior research carried out?, alone?).

- What were the choices?

- How were judgements arrived at?

- What were their expectations of service provision (Prefer to meet the same health professional/ 'one stop shop', or not).

**5) Utilising the service-**

***Aim: how do patients go about using the screening programme.***

- Actions (What do you do when you come to Barts for a clinic appointment?, what happens?, any routine actions?).

- Behaviours of the patient (e.g. any planning, organising, flexibility: pre, during and post appointment).

**6) Overall experiences as a patient using the service-**

***Aim: understanding patients with a certain endocrine disorder lived experiences.***

- What impact (if any) of having the condition does it have? (any emotional difficulties, any effect on daily/ personal/ family life).

- Was it worthwhile? (feel like it adds positive/negative/nothing to life experience).

- The positive and negative dimensions to their experience (interaction with staff/ barriers/ facilitators).

- Reflection (sense of anchorage and safety or uncertainty/ affect outlook on future).

**7) Suggestions for improvements-**

***Aim: to obtain patients’ suggestions for what would improve the*** ***Barts Endocrine Screening Programme and close interview on a positive note.***

- How do you think we can improve patient experiences within these services?

- What matters most to you as a patient?

- Looking back now is there anything specifically that would have made your experience easier/better?

- Anything else you would like to add?

- Any questions you would like to ask me?

**End interview and thank participant.**

**Ensure participant is not distressed/uncomfortable.**
