# Supplementary material for: Factors Affecting Engagement in Screening Clinics; Exploring the Experiences of Patients with Rare Endocrine Gene Disorders
Source: J Patient Exp. 2025 Mar 16;12:23743735251316120. doi: 10.1177/23743735251316120 (PMC11912168; doi:10.1177/23743735251316120)
Supplement: sj-docx-2-jpx-10.1177_23743735251316120 - Supplemental material for Factors Affecting Engagement in Screening Clinics; Exploring the Experiences of Patients with Rare Endocrine Gene Disorders [file sj-docx-2-jpx-10.1177_23743735251316120.docx]

| **Guide questions/description**  **Supplementary material 2**  COREQ (Consolidated criteria for Reporting Qualitative research) 32 item Checklist  Developed from: Tong A, Sainsbury P, Craig J. Consolidated Criteria for Reporting Qualitative Research (COREQ): A 32-item checklist for interviews and Focus Groups. *International Journal for Quality in Health Care*. 2007;19(6):349-357. doi:10.1093/intqhc/mzm042. | **Comment** | **Location in manuscript**  **or not**  **applicable(N/A)** |
| --- | --- | --- |
| **Domain 1: Research team and reflexivity** | | |
| **Personal Characteristics** | | |
| 1. Interviewer/facilitator  Which author/s conducted the interview or focus group? | The lead researcher conducted the interviews | Methods |
| 2. Credentials  What were the researcher’s credentials? E.g. PhD, MD | BSc MSc | N/A |
| 3. Occupation  What was their occupation at the time of the study? | At the time of the study a student researcher | Methods |
| 4. Gender Was the researcher male or female? | Female | N/A |
| 5. Experience and training What experience or training did the researcher have? | Safeguarding level 2 | N/A |
| **Relationship with patients?** |  |  |
| 6. Relationship established  Was a relationship established prior to study commencement? | Due to previous clinical preliminary observations, the researcher was not a stranger to the interviewees, therefore rapport building, which creates a platform and sets the tone for the interview, was already established. | N/A |
| 7. Patient knowledge of the interviewer  What did the patients know about the researcher? e.g. personal goals, reasons for doing the research | Interview patients were briefed on the purpose of the study and understood that it was a research project for Barts  Charity. Ethical approval had been granted, Interview patients reviewed the patient information documentation prior to giving their written informed consent to be involved. | N/A |
| 8. Interviewer characteristics What characteristics were reported about the interviewer/facilitator? e.g. Bias, assumptions, reasons and interests in the research topic | The interviewer was known to the interview patients  which was a potential source of bias. No other interviewer-related biases identified. | N/A |
| **Domain 2: study design** |  |  |
| **Theoretical framework** |  |  |
| 9. Methodological orientation and Theory  What methodological orientation was stated to underpin the study? e.g. grounded theory, discourse analysis, ethnography, phenomenology, content analysis | Constructivist  theoretical approach | N/A |
| **Patient selection** |  |  |
| 10. Sampling How were patients selected? e.g.  purposive, convenience, consecutive, snowball | Purposive | Methods |
| 11. Method of approach  How were patients approached? e.g. face-to-face, telephone, mail, email | Face-to-face | Methods |
| 12. Sample size How many patients were in the study? | twelve | Results |
| 13. Non-participation  How many people refused to participate or dropped out? Reasons? | All the interview patients gave informed consent and completed the interview. There were no patients who subsequently refused to participate, withdrew consent or dropped out. | N/A |
| **Setting** |  |  |
| 14. Setting of data collection  Where was the data collected? e.g. home, clinic, workplace | Data was collected in the location most appropriate for the interview patient. | Method |
| 15. Presence of non-patients Was anyone else present besides the patients and researchers? | Yes, for three of the patients their parents were present. | Method |
| 16. Description of sample  What are the important characteristics of the sample? e.g. demographic data, date | Age range 10-66; 6 males, 6 females. Date was collected between 4^th^ July 2018 to 8^th^ September 2018. | Results |
| **Data collection** |  |  |
| 17. Interview guide  Were questions, prompts, guides provided by the authors? Was it pilot tested? | Interviews were semi-structured using an interview topic guide which included prompts provided. The final topic guide was piloted. However, due to the challenge of accessing participants with rare syndromes, this was done within the supervisors and role play. Interview topic guide is provided as supplementary material 1. | Methods |
| 18. Repeat interviews  Were repeat interviews carried out? If yes, how many? | No | N/A |
| 19. Audio/visual recording  Did the research use audio or visual recording to collect the data? | The semi-structured interviews were audio recorded using a Dictaphone. | Methods |
| 20. Field notes  Were field notes made during and/or after the interview or focus group? | Filed notes were made after the interviews. | N/A |
| 21. Duration  What was the duration of the interviews or focus group? | The semi-structured interview durations ranged from 04:58 to 32:18 (minutes: seconds) | N/A |
| 22. Data saturation  Was data saturation discussed? | During development of themes with the authors. | N/A |
| 23. Transcripts returned  Were transcripts returned to patients for comment and/or correction? | No | N/A |
| **Domain 3: analysis and findings** |  |  |
| **Data analysis** |  |  |
| 24. Number of data coders  How many data coders coded the data? | One (SE) | Data analysis |
| 25. Description of the coding tree  Did authors provide a description of the coding tree? | Codes were collated into categories, sub-themes and finally into candidate themes. | Data analysis |
| 26. Derivation of themes  Were themes identified in advance or derived from the data? | Themes were derived from the data. | Data analysis |
| 27. Software What software, if applicable, was used to manage the data? | NVivo 12 was used | Data analysis |
| 28. Patient checking  Did patients provide feedback on the findings? | No | N/A |
| **Reporting** |  |  |
| 30. Data and findings consistent  Was there consistency between the data presented and the findings? | Yes, specific comments were supported with direct quotes attributed to anonymised interview patient. | Results |
| 31. Clarity of major themes  Were major themes clearly presented in the findings? | Yes main themes are identified | Results |
| 32. Clarity of minor themes  Is there a description of diverse cases or discussion of minor themes? | Yes subthemes are identified | Results |
